# A conceptual framework of indicators for the suitability of forests for outdoor recreation

**DOI:** 10.1007/s13280-024-02091-8

**Published:** 2024-11-23

**Authors:** Carl Lehto, Anders Sirén, Marcus Hedblom, Peter Fredman

**Affiliations:** 1 Department of Ecology, Swedish University of Agricultural Sciences, Box 7044, 750 07 Uppsala, Sweden; 2 Universidad Intercultural de las Nacionalidades y Pueblos Indígenas Amawtay Wasi, Av. Colón E5-56 y Juan León Mera, Edif. Ave María, Torre B, Quito, Ecuador; 3 Department of Urban and Rural Development, Swedish University of Agricultural Sciences, Box 7012, 750 07 Uppsala, Sweden; 4 Department of Economy, Geography, Law and Tourism (EJT), Mid Sweden University, 831 25 Östersund, Sweden

**Keywords:** Fennoscandia, Forests, Indicators, Indices, Outdoor recreation, Recreation potential

## Abstract

Forests’ ability to provide opportunities for recreation is an important ecosystem service. This has prompted attempts to create indicators to assess forests' suitability for recreation, although hitherto with limited success. This study introduces a novel framework for indicators of potential and realised recreational values of forests, with a primary focus on Sweden and Fennoscandia. We divided forest attributes into intrinsic qualities (i.e. the structure and composition of the forest), extrinsic qualities (i.e. the location of the forest in relation to other components of the landscape), and facilitation qualities (i.e. the presence of recreational infrastructure). Using Fennoscandia as a case study, we performed a literature review to find specific indicators of recreational values, as well as evaluate the current availability of spatial data suitable to map the forest qualities on a national scale. The most important intrinsic quality we identified was tree size/age, whereas for extrinsic quality it was proximity to water. Systematic monitoring of recreational use is essential to estimate realised recreational values. The conceptual framework proved to be a valuable tool for identifying potential indicators, and applying it in other regions is likely to yield useful outcomes.

## Introduction

Forests provide important opportunities for outdoor recreation. In Europe, seventy percent of forests are available for public recreation, and about six percent are primarily designated or managed for public recreation (FOREST EUROPE 2020). Forests contribute to attractive living spaces, nature-based tourism opportunities, and improved public health (Bell et al. 2009). Hence, recreation in forests and other green spaces delivers significant ecosystem services (IPBES 2019) which need to be recognised, measured and quantified to receive more attention in policy and management decisions (Pohjanmies et al. 2017; Schägner et al. 2018). However, quantifying the value of this ecosystem service poses challenges, which have made recreation of secondary importance in physical planning (Petersson-Forsberg 2014) and forest management decisions (Angelstam et al. 2020). This has led to a decrease in the amount of accessible greenspace (Richards and Belcher 2020).

While forests worldwide provide a variety of recreation opportunities, the trends and drivers behind outdoor recreation activities are becoming increasingly diversified with different demands on forest attributes (Edwards et al. 2012a, b; Elmahdy et al. 2017; Giergiczny et al. 2015; Manning et al. 2022). This increases the difficulty in integrating recreational values into forest planning systems, necessitating robust models capable of including larger varieties in user preferences across forest regions and recreation activities. In order to achieve this, appropriate and efficient indicators are paramount (Nordic Council of Ministers 2013). Previous attempts have been made to identify indicators that can be used to create indices of recreational potential across entire landscapes based on people's preferences (e.g. Komossa et al. 2018; Paracchini et al. 2014; Peña et al. 2015; Walz and Stein 2018). Such spatial indices have primarily used extensive scales, spanning from the entire EU (Komossa et al. 2018; Paracchini et al. 2014) to countries or regions (Peña et al. 2015; Walz and Stein 2018). Komossa et al. (2018) estimated recreational potential for five “archetypical” user groups with different preferences, whereas other sets of indicators have primarily been based on landscape attributes presumed to be universally preferred, such as proximity to water or a higher “naturalness”, although there is no consensus about how to actually define and measure the latter (Winter 2012).

At the European level, The Ministerial Conference on the Protection of Forests in Europe (FOREST EUROPE 2020) has noted that although spatial indices have greatly enhanced our comprehension of recreational area availability, this broad approach is often too coarse to be effectively applied in local physical planning and management of forests. Similarly, a compilation of experiences from nine northern European countries on the variations in index design and application highlighted that while some indirectly reflect social forest values, few directly gauge the demand or supply of recreation (Nordic Council of Ministers 2013). This could be attributed to the fact that indicator selection is often guided by data availability, and the available data does not necessarily accurately capture key recreational values. On-site data of recreational use and preferences is paramount to evaluate the demand for outdoor recreation opportunities, but also for providing measures for multifunctional forest management (Schägner et al. 2018). Thus, there is a need for better knowledge of recreation indicators taking to account both actual recreation possibilities and perceived possibilities.

Elaborating useful indicators is also hampered by a lack of a suitable conceptual framework for the phenomenon in focus (Nordic Council of Ministers 2013; Sievänen et al 2013). Whereas some studies highlight, e.g. the importance of ecological characteristics others highlight different aspects of accessibility, recreationists’ perceptions, or just counting visitors. A conceptual framework that clarifies how the results of studies using such distinct approaches relate to each other has been lacking.

The primary objective of this study is to develop a robust conceptual framework for indicators of recreational values in forests which can be universally applied. To illustrate the framework we use Sweden and its Fennoscandian neighbours as the case, proposing a relevant set of indicators for assessing recreational values within this specific natural and societal context.

## Methods

### Literature review

We performed a scoping study of relevant literature (Arksey and O’Malley 2005), starting with literature on recreational use of forests that we were already familiar with, combined with searches in Scopus, Google Scholar, and Web of Science. Search terms used were combinations of “Recreation”, “Forest”, “Boreal”, “Temperate”, “Indicator” and “Preference” etc. We employed a snowball methodology, where based on the relevant publications we found (especially review papers), we explored the literature cited in those publications, and used the databases to search for more recent publications cited in each publication.

We found three review articles on visual aesthetic values of landscapes in general (Bishop 2019; Freimund et al. 1996; Lothian and Bishop 2017), and three on forests landscapes specifically (Ribe 1989; Gundersen and Frivold 2008; Gundersen et al. 2019). We also found four reviews on the relation between forest characteristics and people's health and well-being (Bratman et al. 2019; Doimo et al. 2020; Grilli and Sacchelli 2020; Gobster et al. 2022).

### Creating the conceptual framework and set of indicators

**Fig. 1:**
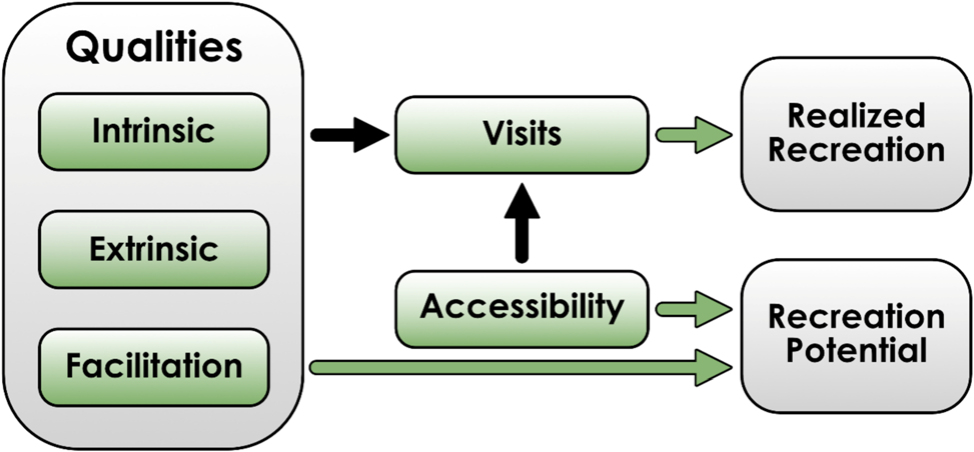
The conceptual framework of how recreation visits to a forest are driven by its qualities and accessibility (black arrows). Realised recreational values can be estimated by measuring the number and quality of forest visits by users, while the recreation potential of a forest can be assessed by using the forest’s qualities and accessibility as indicators (green arrows)

Creating a conceptual framework for recreational values of forests (Fig. 1) was an iterative process. After having gone through a large amount of literature, we created a preliminary framework where we tried to fit, in a coherent manner, all the different characteristics that, according to different studies, have been shown to be important for the recreational values of forests. When we found incoherencies in the framework, we modified it, until it got its final form. Whereas this conceptual framework should be applicable to forest recreation in basically any part of the world, the indicators we then suggested were chosen specifically with the case of Sweden and its Fennoscandian neighbours in mind. Sweden has a larger forest area than any other EU country, holding 18% of the union’s forest, with Finland not far behind. Forests in Sweden, Finland and Norway are ecologically similar to each other, consisting mostly of boreal forests dominated by conifers, with some deciduous forests in the southern region and near the treeline at high altitudes. Also the cultural context is similar in the three countries, and visiting forest is a very common leisure activity. National surveys show that between 75 to 90 percent of the adult population visit forests annually (Sievänen et al. 2013), numbers which rose dramatically during the COVID-19 pandemic (Hedenborg et al. 2022). Most of the forests are managed for timber production, with a typical management cycle being clearcutting, followed by planting and subsequent thinnings. Only eight percent of the forests are legally protected from logging (Angelstam et al. 2020), hence much recreation occurs in forests whose primary function is to produce timber.

The choices of indicators to include in the set were based primarily on research from the Fennoscandian region, but we also included research from other regions when deemed applicable. Two of the reviews we found focused specifically on Fennoscandian forests (Gundersen et al. 2019; Gundersen and Frivold 2008). We also found two previous attempts to create spatial indices for boreal forests in Sweden. Olsson (2013) used parameters from previous photo studies on forest stands to classify forests as suitable or unsuitable for recreation based on available forest map data in order to estimate the change in area of forests suitable for recreation near urban areas. Another approach was found in the Heureka forest decision support system, a computer program for forest management planning (Wikström et al. 2011). Heureka includes three recreation indices for forest stands, where the age of the forest stand determines which to apply. These indices are also based on results from previous photo studies and require detailed data at the stand level.

To evaluate which forest qualities were relevant to include as indicators we employed two criteria: (i) The robustness of research showing the relation between the quality and recreational preference; and (ii) the current availability in Sweden (or feasibility of production) of spatial data that captured the quality on a national scale.

One complication of the first criteria is that preferences vary (Abildtrup et al. 2013; Giergiczny et al. 2015; Manning et al. 2022). A classic article in outdoor recreation research with the title “The Average Camper Who Doesn't Exist” effectively illustrates the problem of designing management strategies for recreation areas based on an "average visitor" (Shafer 1969). Studies have shown that individuals exhibit a variety of preferences and environmental needs in the context of outdoor recreation: for instance, Kienast et al. (2012) uncovered distinct preferences among older individuals, while Elbakidze et al. (2022) and Eriksson et al. (2012) demonstrated that older people, women, and those with higher levels of education value urban green spaces more. However, socio-demographic attributes seem to possess limited explanatory power in deciphering people's inclinations towards natural environments compared to environmental attitudes, nature-relatedness, or ideological stances (Scott et al. 2009; Eriksson et al. 2012; Ode Sang et al. 2016; Juutinen et al. 2017). There are many drivers behind recreational demand in the social, technological, economic, environmental and political domains (Elmahdy et al. 2017). There is, for example, evidence that the cultural context can have an effect, with Edwards et al. (2012a, b) recognising some regional differences across Europe for which forest characteristics were preferred (see also Pelyhukh et al. 2019). Stronger effects seem to emanate from the type of recreational activity engaged in, especially in connection to what facilitation qualities are preferred (Kienast et al. 2012; de Valck et al. 2016, 2017; Korpilo et al. 2017).

When identifying potential indicators, we primarily focused on characteristics that are generally perceived similarly by most people. However, in certain cases, especially regarding recreational infrastructure, there is significant diversity in preferences. This heterogeneity cannot be ignored and must be integrated into the indicators.

## Results

### A conceptual framework of forest recreational values

The conceptual framework we propose (Fig. 1) posits that individuals’ decisions to visit forests for recreation are influenced by two main factors: *accessibility* and the forest’s *qualities* (Sievänen et al. 2008).

*Forest qualities* are place-bound attributes of the forest, which we divide into three categories: First are the *intrinsic* qualities, encompassing physical attributes of the forest such as its structure and species composition. Second are the *extrinsic* qualities, referring to attributes related to the forest's surroundings, such as topography or proximity to other landscape features. Third are the qualities created through *facilitation*, e.g. the presence of recreational infrastructure such as paths, bridges, benches, toilets, fireplaces, information boards, or other amenities that facilitate recreational activities.

*Accessibility* refers to how easily users can access the forest. There’s a large body of evidence that this is a critical aspect that shapes where recreation occurs (Hörnsten and Fredman 2000; Grahn and Stigsdotter 2003; Agimass et al. 2018). Accessibility is largely a feature of the physical landscape (e.g. distance, barriers, infrastructure), but also has a cultural, social, and socio-psychological dimension related to attributes of the observer such as knowledge about a forest and the recreation opportunities, sense of safety, and previous experiences, which interact to create a perceived landscape accessibility (Koppen et al. 2014). In our conceptual framework, the physical dimension of accessibility refers exclusively to *external* accessibility, meaning how easily a user can reach the point where travel ends, and recreation begins. *Internal* accessibility within a forest (such as trails) is treated separately through the forest qualities.

The framework further defines *realised recreation* as the ‘true’ recreational value of a specific forest or of a specific recreationist. This can be estimated by using the quantity of visits and/or the experienced satisfaction as indicators. Conversely, *recreation potential* is a forest’s theoretical attractiveness for recreation, regardless of whether it is currently being used for recreation or not. This aspect can be estimated by using accessibility and the qualities as indicators. Estimating *realised recreation* leads to results that are closer to the ‘real’ recreational value of a forest, but is impractical for entire forest landscapes, and also fails to identify forest areas that have a potential to attract recreational users but currently does not do so, e.g. because they are unknown to the public. This is why it is important to also estimate the recreation potential of forests.

### Intrinsic forest qualities

In the literature review, we identified six qualities that significantly affect the recreation potential of a forest: Tree size/age, stand density/visibility, traces of forestry operations, stand heterogeneity, tree species composition, and biodiversity.

#### Tree size/age

The presence of large or old trees have consistently been shown to yield a positive response in preference studies (Gundersen and Frivold 2008; Gundersen et al. 2019) and estimated to be the most important quality by experts (Edwards et al. 2012a, b). The presence of large or old trees was also a common feature of forests used for forest therapy (Gobster et al. 2022). Conversely, young forests consistently yield low preferences (Gundersen and Frivold 2008; Edwards et al. 2012a, b).

Age, height and diameter of trees are highly correlated with each other, and the literature does not clearly show which of them is the decisive factor. An indicator relying on tree height alone would make forests in the south of Sweden appear generally more attractive for recreation than forests in the north, where the climate is harsher. Similarly, forests on rocky outcrops, windy shorelines, or nutrient-poor peatlands, where trees never grow very high, would also yield low scores. To resolve this we therefore suggest an indicator based on mean tree height weighted by basal area, normalised against the forest *site inde*x, which is the maximum height trees can attain at the site in question at a defined reference age. At present, the best available map data source is the Swedish Forest Agency's map service *Skogliga Grunddata*, which can estimate tree heights with 12.5 m spatial resolution (Swedish Forestry Agency, n.d.). Maps of forest site index, with national coverage, are currently in development (Mistra Digital Forest, n.d.).

#### Stand density and visibility

High stand density is generally perceived negatively, both because of the perceived low accessibility, with a dense forest being more difficult to pass through, and because of the perception of the depth of visibility. Long sightlines and large vistas are generally attractive traits in landscape studies, but visibility within a forest can only be increased up to a certain limit—at some point the feeling of being in a forest ceases. The literature provides little guidance on where this limit is. Regardless, intermediate stand densities and visibility are preferred, whereas the extremes—forests that are either too dense or too sparse—are perceived as less attractive (Gundersen et al. 2019).

A further complication is that depth of visibility has no clear definition and is difficult to capture via map data. Previous attempts have created indicators from basal areas of trees to capture this aspect (Olsson 2013; Wikström et al. 2011). However, the link between basal area and perceived density is probably weak, as visibility is mainly reduced by young trees which have dense branches near the ground, but only have a small basal area. Forest density and visibility depth could potentially be estimated by using LiDAR data (Zong et al. 2021), which is now available for the entire area of Sweden. Such methods need however to be calibrated for the area they are employed in, and would also be computationally demanding to implement on a larger scale.

#### Traces of forestry operations

Fresh traces of forestry operations, such as stumps, logging residues or ground damage from machinery are perceived negatively by most people (Gundersen et al. 2019). The effects seem to be correlated to the intensity of the operation, with clearcutting yielding the strongest negative reactions. Mattsson and Li (1994) showed that recreational values could be increased through a decrease in clearcutting with artificial regeneration in favour of natural regeneration, as well as a reduction of spruce, in favour of broadleaved trees. These effects seem to be modulated by the background of the person, with people who have a background in forestry more positive to typical forest management operations (Kearney and Bradley 2011). Traces of forestry operations are not currently possible to capture in a spatial indicator on a national level due to a lack of available map data, except for clearcuts. However, since such forestry measures also directly affect the presence of large/old trees, this aspect is partially captured through the inclusion of that quality.

#### Stand heterogeneity

There is evidence that heterogeneity is a preferred aesthetic trait (Kaplan and Kaplan 1989; Dronova 2017). The concept is scale-dependent, e.g. different forest stands could be experienced as having various degrees of heterogeneity, but also an entire forest could be perceived as being more or less heterogeneous. In this section, we focus on the stand level, whereas landscape level heterogeneity is covered under *Extrinsic forest qualities* below.

In the few studies focusing specifically on forest heterogeneity, there is some evidence for positive effects. In a Danish study, participants preferred forests with variation of tree heights and species composition, both within and between stands (Filyushkina et al. 2017). Pelyhukh et al. (2019) showed that forest stands with a random mix of diameters were preferred. In a study where expert panels from four European regions were asked to rank which forest characteristics were most important, variation between forest stands was ranked as number 8 out of 12 alternatives (Edwards et al. 2012a, b). A heterogenous species composition was also positively linked to preference in a UK study (Tew et al. 2019), and was one of the most common features mentioned in a review of studies on forest therapy (Gobster et al. 2022). Variation thus appears to contribute positively to the recreational value of forests, but there is currently no consensus on how to define and measure it.

#### Tree species composition

Conifers dominate most forests in Sweden. This is partly a natural phenomenon as Norway spruce (*Picea abies*) and Scots pine (*Pinus sylvestris*) are the most common native tree species in the country, but in addition, silviculture operations have favoured conifers over deciduous species. Deciduous trees make up a smaller fraction of the forest (~ 20%), with the main species being birch *Betula pendula* and *B. pubescens*. Except for the mountain birch forests near the tree line in the mountains, only scattered patches remain of forests where deciduous trees dominate. Preference studies do not provide a clear picture of which species are preferable for recreationists, but most seem to indicate that mixed forests that include deciduous species are preferred (Filyushkina et al. 2017; Gundersen et al. 2019). It is not clear whether this effect stems from a preference for deciduous trees, or for the increase in perceived heterogeneity that deciduous trees provide in otherwise coniferous-dominated forests. In Sweden, nationwide map data on the proportion of tree species is available via Skogliga Grunddata (Swedish Forestry Agency, n.d.).

#### Biodiversity

Studies on how recreational preferences relate to biodiversity has yielded mixed results and few of them have focused specifically on forests. Some have demonstrated a positive impact on individuals' self-reported well-being in environments with higher species richness (Fuller et al. 2007; Gunnarsson et al. 2017; Wood et al. 2018; Cameron et al. 2020), whereas others have showed no significant effect (Dallimer et al. 2012; Qiu et al. 2013). In Finland, national parks with higher numbers of red-listed species attracted more visitors, yet this was also linked to the diversity of Natura 2000-habitat types (Siikamäki et al. 2015). A similar trend is evident in a UK study focusing on bird diversity, habitat diversity, and well-being in urban green spaces (Cameron et al. 2020). As habitat diversity and species richness tend to correlate positively both with each other and with recreational preference, it is unclear which of the two is the crucial factor for recreationists (Fuller et al. 2007). There is also quite some variation between subjects, as individuals with a strong affinity for nature tend to derive more enjoyment from biodiversity than those less nature-oriented (Gunnarsson et al. 2017).

The presence of deadwood, which is important for forest biodiversity, yields mixed responses. Some earlier studies showed low preference for forests with substantial amounts of deadwood (Gundersen and Frivold 2008), whereas more recent investigations suggest that attitudes towards deadwood have improved, possibly because of increased awareness of its importance for biodiversity (Heyman 2012; Hauru et al. 2014; Gundersen et al. 2017).

Biodiversity cannot be captured by a single metric. Research on its impact on recreational preferences has often focused on *species richness* of specific taxonomic groups (mainly vascular plants, birds, and butterflies)—which is only one facet of biodiversity. However, the applicability of findings from studies concentrating on certain groups and indicators to other taxa and dimensions of biodiversity remains uncertain. This complexity, coupled with data limitations, makes it impractical to integrate biodiversity as an indicator of recreational value in forest environments.

### Extrinsic forest qualities

In the literature review we identified four qualities related to a forest’s location and surroundings that affect recreational value: proximity to water, the access to scenic views, the presence of noise, and landscape heterogeneity.

#### Proximity to water

There is a substantial body of research showing that there is a general, strong preference for aquatic environments (Kaplan and Kaplan 1989; White et al. 2010), and that this preference translates to increased visitation rates (Kienast et al. 2012). The presence of water was the most common characteristic of forests used in forest therapy studies (Gobster et al. 2022), and there is strong evidence for the link between spending time near water and human well-being (White et al. 2020). The few studies made about this issue specifically in Fennoscandia show a similar picture. In Sweden, forests that had elements of water were shown to more effectively instil a sense of recovery than other forests (Sonntag-Öström et al. 2011).

Proximity to water was included as an indicator in the Heureka model for forest planning, assuming that all forest stands within 50 m of water bodies had higher recreational values (Wikström et al. 2011). Olsson (2013) instead assumed that the recreational value of water decreased linearly with distance, lakes being assumed to have an effect up to 500 m distance, whereas streams and rivers were assumed to have an effect up to 100 m. The literature does not fully address to what extent characteristics such as walking distance, visibility, or the size and type of the water body, matter.

#### Noise

Exposure to noise has been recognised as a public health problem (Basner et al. 2014). Most studies of human health and noise have been conducted in urban settings or green urban proximate settings (Evensen et al. 2016; Fang et al. 2024), with fewer studies conducted on recreation and noise in rural forested areas. However, a study from the United States found that 63% of all protected natural areas had a noise level twice as high as the natural due to anthropogenic sources, and 21% of them had a tenfold increase (Buxton et al. 2017).

We did not find extensive literature on the connection between recreation and noise in Swedish forests, but a national survey showed that 23% of recreationists experienced noise during their latest visit to a forest (Swedish Environmental Protection Agency 2015). A Norwegian study on recreational values before and after the relocation of an airport compared how recreationists experienced the forests around the old and new airports before and after the move (Krog et al. 2010). Unsurprisingly, recreational values around the old airport improved when flights ceased, whereas they deteriorated around the new airport. In Sweden, data on industry, railways and roads have been used to generate nationwide map data of estimated noise levels (Jönköping Administrative County Board 2015).

#### Topography and views

There is a strong general preference for places that provide scenic views (Kaplan and Kaplan 1989), thus the topography of a forest area can increase recreational values. Visible objects can also have an impact; negative aesthetic effects on the landscape are for example often raised as an argument against wind turbines (Dai et al. 2015). In addition to scenic views, such topographical features as cliffs and ravines can be popular destinations and sometimes a requirement for recreational activities like climbing. Similar to stand density, views can be assessed through LiDAR-based visibility analysis, but for computational reasons this is currently impractical to do on a large scale. A simpler approach could be to use topography to increase the recreational value of forest stands that are situated higher than the surrounding areas. There is currently no data available that allows for large-scale mapping of visually disturbing features such as built-up areas or wind turbines. To some extent, however, as such features also tend to generate noise, the inclusion of the national noise map (see Sect. 4.2.2) would to some extent also capture areas where visually disturbing features impact the recreational value of forests.

#### Landscape heterogeneity

Heterogeneity has a positive effect on recreational preferences not only within forest stands, but also on a landscape scale (de la Fuente de Val et al. 2006; Dramstad et al. 2006; De Valck et al. 2017; Dronova 2017; Hahn et al. 2018; Tew et al. 2019). There is however no consensus on how to define or measure landscape heterogeneity. Previous attempts have used various information indices (e.g. Shannon–Wiener index, Simpson’s index) using land cover classes (Dronova 2017). This approach has been criticised because the link to the actual perception of heterogeneity is weak (Cale and Hobbs 1994). There are ongoing efforts to improve indicators of landscape heterogeneity (e.g. Díaz-Varela et al. 2016) but currently challenges still remain.

### Facilitation

There is a long tradition of providing various types of infrastructure to facilitate recreation opportunities, such as trails, roads, signs, shelters, public toilets, and visitor centres in recreational areas (Haukeland et al. 2014; Tverijonaite et al. 2018). Today, infrastructure also extends into the virtual realm, in the form of websites and apps (Muñoz et al. 2019; Chekalina et al. 2021). There is clear evidence that this type of facilitation is important for recreation, with correlations between presence of recreational infrastructure and preference and/or number of visits (Kienast et al. 2012; Giergiczny et al. 2015; Donovan et al. 2016; de Valck et al. 2017). In a Norwegian study, respondents were asked to look at photographs of trails with different levels of preparation (from nature trails to paved footpaths) combined with measuring the number of actual visits to similar trails (Gundersen and Vistad 2016). The results showed an interesting paradox; participants expressed higher preferences for more natural trails, while the frequency of visits showed the opposite, with more prepared trails having a higher usage.

Although many people appreciate the presence of recreational infrastructure, there is a high degree of preference heterogeneity, recognised in recreation studies using the so-called “wilderness purism scale” as well as in planning frameworks such as the Recreation Opportunity Spectrum (ROS, Fredman and Emmelin 2001; Manning et al. 2022; Sæþórsdóttir et al. 2022). This scale suggests that recreationists are situated on a spectrum between more urban oriented visitors ("urbanists"), who seek easily accessible and comfortable nature, and the more wilderness oriented visitors ("purists") who seek solitude and challenges. A central tenet of the ROS framework is that the planning should provide good recreational environments for a spectrum of different visitors with different preferences.

Given that facilitation seems to be of crucial importance for the recreational experience, we suggest that this should be included as an indicator, but the heterogeneity of preferences need to be accounted for. This could be done by modifying the recreation potential index for different user groups, similar to the approach in Komossa et al. (2018). Currently, however, there is a lack of data on recreational infrastructure that makes it difficult to include it as an indicator on a national level; there is systematic coverage only within formally protected nature and recreation areas (Swedish Environmental Protection Agency, n.d.). Open Street Map^1^ contains data on paths and recreational infrastructure in general, but as it is largely based on voluntary contributions, it is likely to contain considerable biases. Given the general trend of increasingly specialised outdoor recreation activity patterns (where facilities play an important role), preference heterogeneity is likely to become more important to consider in future forest management.

### Realised recreation versus recreation potential

In addition to the forest qualities, the conceptual framework also distinguishes between *realised recreation* vs. *recreation potential*. The number of visits to a particular forest can be seen as a “realised” measure of recreational value. If people had perfect knowledge about recreation opportunities in different forests, and no cost of getting there, visit rates would in theory perfectly reflect their intrinsic, extrinsic and facilitation qualities. In real life, however, nobody has perfect knowledge, there are various types of costs associated with forest visits, and several other factors constrain outdoor recreation (Jackson 2005; Fredman et al. 2011). Knowledge about forest visits and visitors are therefore paramount to successful management of forests for recreation.

#### Realised recreation

Two types of data are needed to measure realised recreation: visitor numbers and the outcomes/impact that follow from a recreation visit (Kajala et al. 2007; Ankre et al. 2016 ). People decide to recreate in a certain location based on forest characteristics and after “negotiating” their personal motivations, benefits, and constraints (Jackson 2005; Manning 2022). Visits to nature will result in different types of outcomes (for individuals and societies) and often involve different types of impacts (economic, environmental and/or social). In addition, there are also people not currently recreating in forests that might decide to do so in the future, hence every forest also has an option value to deliver recreation services in the future.

In practice, there has been more focus on measuring the frequency of visits, and less on the quality of the recreationists’ experience (Kajala et al. 2007). This might be partly due to the former being easier to measure than the latter, but also due to a belief that high visit frequency in an area reflects high quality experiences. However, as recreational usage is closely correlated with accessibility to nearby areas used more frequently, the link between visit frequency and quality can be rather weak (Lehto et al. 2022). Hence, it is therefore important to incorporate measures of quality and satisfaction in outdoor recreation monitoring (Manning et al. 2022). Forest planning and management should consider the different demands for forest qualities (Gundersen and Frivold 2008; Mattsson and Li 1994; Ode Sang et al. 2016) and needs for recreational infrastructure (Gundersen and Vistad 2016). With new digital technologies this has become easier to implement (Muñoz et al. 2019; Heikinheimo et al. 2020). Methodological improvements include further developments of traditional methods such as digital surveys with GIS elements (PPGIS), but also new ones such as mobile phone tracking, data scraping of social media and the use of smartphone applications. These technologies have the potential to collect visitor data with higher accuracy and resolution, with less effort from participants and at a lower cost, however they also come with limitations in terms of sampling bias, cooperation and privacy (Miyasaka et al. 2018; Muñoz et al. 2019).

The measurement of forest visits, visitors, and associated outcomes typically call for a combination of visitor counting and survey data (on site as well as from general population surveys) collected systematically over time (Kajala et al. 2007). We advise forest managers to take inspiration from visitor monitoring approaches used in protected area management (Ankre et al. 2016; Pickering et al. 2018).

#### Recreation potential

To implement the suggested set of indicators into a measure of recreation potential, a couple of critical aspects need to be considered. The first is the spatial scale on which the measure is applied. Olsson et al. (2013) used the scale of the map data (25 × 25 m), and classified each forest patch. This approach, although simple to execute, has its pitfalls: a recreational experience flows over multiple scales, and walking through a forest is not simply the sum of a number of separate forest stands. Certain qualities are reasonable to estimate on such a scale, while others (e.g. landscape heterogeneity) necessitates a larger perspective.

A second consideration is what the output of the measure is. One possibility is to classify each forest as either suitable or unsuitable for recreation, e.g. as in Olsson et al. (2013). With such an approach a reasonable weighting of the indicators might be difficult to achieve: Some qualities can immediately disqualify a forest as suitable for recreation (e.g. a recent clearcut), while others rather modulate its recreational value (e.g. proximity to water). The alternative is to implement a continuous scale, which is the more common approach (e.g. Paracchini et al. 2014; Komossa et al. 2018).

With these considerations solved, a set of nationwide raster maps representing the recreation potential of forests could be created by combining the various map data mentioned above. These raster maps of estimated recreation potential can then be compared to georeferenced population data to calculate (1) forest-centred recreation potential, i.e. the recreation potential of different forests; (2) the user-centred recreation potential, i.e. the amount of high quality forests accessible for each user. It is possible to motivate certain distances of "accessible distance” from literature such as, e.g. 250–300 m (Grahn and Stigsdotter 2003), 1 km (Hörnsten and Fredman 2000) or 2.5 km (Suárez et al. 2020). The simplest approximation of accessibility is using straight-line distances, a more realistic alternative is to employ network analysis which calculates travel time based on the presence of the road network as well as presence of barriers, such as rivers etc. (e.g. Albacete et al. 2017).

### Selecting indicators

In summary, we identified six intrinsic qualities, four extrinsic qualities and facilitation (composed of many possible elements) to play a significant role for the recreational value of forests (Table 1). Tree size/age is one of the most important indicators of intrinsic quality, whereas proximity to water is the most important extrinsic quality. Regarding facilitation, the presence of recreational infrastructure would be the suggested indicator, possibly divided into several sub-indicators, e.g. paths, shelters, signage etc. The coverage of data on a national level is as mentioned however restricted to protected areas such as national parks and nature reserves. Since this quality is subject to a higher degree of preference heterogeneity, it needs special considerations when implementing.

**Table 1:** Forest qualities identified in our literature review as important for recreational value

Intrinsic qualities	Significance	Current feasibility
Tree size/age	+++	+++
Stand density/visibility	++	+
Traces of forestry operations	+++	
Stand heterogeneity	++	+
Tree species composition	+	++
Biodiversity	+	
*Extrinsic qualities*		
Proximity to water	+++	+++
Noise	++	++
Topography and views	++	+
Landscape heterogeneity	++	+
*Facilitation qualities*		
Recreational infrastructure	+++	++

“Significance” represents the strength of the connection between the quality and recreational preferences. “Current feasibility” shows how applicable we assess the quality to be as an indicator, weighing the combined significance with current data availability and methods to estimate the quality. The number of “ + ” indicates the estimated “strength” of each parameter

Moreover, the conceptual framework of realised recreation and recreation potential can be implemented using two perspectives, *user-centred* and *forest-centred*, which results in a two-dimensional matrix (Table 2). The former evaluates the recreation potential available to a single recreationist as well as the recreation actually realised by them. A forest-centred approach instead assesses the potential of a specific forest to provide recreation to users, as well as the recreation actually realised in that forest.

**Table 2:** Implementing the conceptual framework in a user-centred or forest-centred perspective

	The forest	The user
Realised recreation	Frequency and perceived experience of visits to a specific forest	Frequency and perceived experience of visits to forests for a specific user
Recreation potential	The recreation potential of a specific forest given its qualities and accessibility	The recreation potential available to a specific user given the qualities and accessibility of forests

## Discussion

We set out to formulate a proposal for a robust, universally applicable framework for indicators of recreational values in forests. We then subsequently applied this to the case of forests in Sweden and its Fennoscandian neighbours, proposing a set of indicators that could be used to assess recreational values in this particular natural and societal context. Based on these indicators, it should be possible to produce a recreation potential index, capable of producing nationwide map layers showing a) where forests important for recreation are located, and b) to what extent people living in different places have access to forests suitable for recreation. Our proposed method for mapping the recreational values of forests draws on a substantial body of empirical studies on people's recreational preferences and utilises publicly available spatial datasets. The proposed set of indicators still needs further refinement, but once operationalised it can become a valuable tool, effectively integrating the recreational value of forests into land use planning and policy.

The qualities we have identified differ in terms of the ease with which they can be influenced. The intrinsic qualities take a long time to develop, but can be rapidly damaged by, for example, forestry operations carried out by landowners. Positive extrinsic qualities, such as proximity to water or topographical/geological features, are more resistant to change. Negative extrinsic qualities, like noise or visually disturbing features, can be altered, but this is often outside the control of the landowner. Facilitation, on the other hand, is more easily modifiable but often depends on public resources. The construction of marked footpaths, benches, signs, and similar infrastructure can be implemented quickly at a relatively low cost, and can also easily be removed without leaving significant traces.

Important intrinsic qualities are the presence of large or old trees, an intermediate stand density/visibility, absence of traces of forestry operations, local heterogeneity, mixed species composition, and possibly, high biodiversity. Prevailing forest management practices in Fennoscandia often counteract such positive qualities by primarily focusing on maximising revenues from timber production. Few trees are left to grow old due to recommended silvicultural practices based on clearcutting with relatively short rotation periods. Clearcutting results in a dramatically reduced recreational value, which recovers slowly. Logging operations using heavy machinery leave deep tracks, and planted forests often consist of homogeneous stands of trees with the same age, size, and species, lacking biodiversity. This implies that there is a high degree of correlation between the different intrinsic qualities identified as important for the recreational potential of forests and forest management. Absence of forestry operations leads to better outcomes on most of the identified intrinsic qualities. Despite this negative relation between forestry operations and recreational potential, also forests that have been subject to forestry operations can hold significant recreational values. In fact, few, if any, of the studies we reviewed on intrinsic qualities and recreational potential of forests were conducted in primary forests; most had at some point been subject to logging, although not necessarily clearcutting. Forest management practices could be adapted to better preserve the recreational potential of forest without abandoning timber extraction altogether.

The literature review revealed that there are some contradictory results and some significant knowledge voids in the field. We found, for example, that the recreational value of forests in many aspects is directly related to naturalness and the absence of forestry operations, but there are also some research findings that contradict this, particularly the often expressed negative attitude to the presence of deadwood (Heyman 2012). It is difficult to draw firm conclusions about these issues because few studies have examined the recreational values of truly old growth natural forests. Those that have done so have been based on a selection of photographs chosen subjectively, without an elaborate strategy to ensure their representativeness. To fully understand what advantages or disadvantages it may have for recreational values to leave forests unmanaged, more extensive studies comparing natural forest landscapes with production forest landscapes are needed.

Another neglected aspect is the effect of forest size. Often one hectare has been considered the minimum for a green space to have a tangible value (Annerstedt van den Bosch et al. 2016), and it has been shown that large forests are preferred to small forests (Agimass et al. 2018; Suárez et al. 2020). The areal extent of a forest ought to be included in the set of indicators, but more research is needed to be able to quantify its impact on recreational value in a meaningful way.

A forest visit typically involves moving through the landscape, experiencing different types of forest as well as other landscape elements. Most studies on recreationists’ forest preferences, however, are based on snapshots in space and time of a specific place, typically based on visual impression alone. Visual impressions constitute an important part of the experience of being in the forest, and preferences in photo studies have been shown to be in agreement with field studies (Silvennoinen et al. 2022), but such studies fail to take into account the effect of landscape heterogeneity. It is also important to take into account the heterogeneity of recreationists. Every single forest cannot satisfy all demands, but at the landscape level a spectrum of different recreation opportunities can be provided to accommodate for diverse preferences (see Manning 2022).

A common weakness of studies on recreationists’ preferences is the representativeness of the sample of subjects. Gundersen et al. (2008) noted that, in most cases, it is not clear which population segment the subjects are intended to represent. Only in very few cases, a random sample of subjects, representing the entire population of the nation, has been used (e.g. Jensen and Koch 2000). In addition, almost all studies are based on adult subjects only, in spite of that spending time in forests is very important for children (Oppliger et al. 2019; Taye et al. 2019), Children, moreover, use forests for different activities than adults and seem to particularly want a varied forest with opportunities for many different types of play (Rydberg and Falck 1998).

We suggest that some of these research shortcomings could be met through the deployment of studies of realised recreation, using revealed preference approaches where people's actual landscape usage is studied. Currently, there's a lack of systematic nationwide data on people's visits to nature in Sweden except for certain urban forests, nature reserves, and national parks. National statistics on outdoor activity participation has been collected since the 1970s, but these data are limited in scope and do not include experiential values. Proposals for systematic visitor surveys have been developed on several occasions, and have been the subject of government commissions on outdoor life, protected nature and rural development, but have never been implemented (Nordic Council of Ministers 2013). A way forward to collect better data could be collaboration between authorities and organisations with the common task of promoting people's opportunities to recreate in nature. Finland has made significant progress in systematic visitor monitoring since the early 2000s (Kajala et al. 2007), and many other countries conduct visitor surveys integrated with management of recreational areas (Pickering et al. 2018). There is a pressing need for more systematically collected data about visitor frequency and experience values in forests. While much of the foundation is in place, challenges lie in resource allocation, political will, and collaboration among relevant authorities and organisations.

## Conclusions

The conceptual framework presented in this study allowed us to categorise different kinds of forest attributes into separate categories, distinguishing *accessibility* from forest *qualities*, and subdividing the latter further into *intrinsic*, *extrinsic*, and *facilitation* qualities. This framework should be applicable to forest planning and management also beyond Sweden and Fennoscandia which we used to create the set of indicators. An ideal setting for forest recreation in this region is where there are large old trees, it is quiet, and there is some river, lake, or seashore nearby. It is relatively straightforward to identify, based on spatial data currently available to the public, areas that have these characteristics, or that have a potential to develop them over time. In addition, systematic monitoring of visitor use and preferences provides knowledge to understand variations in user preferences, which allow for more efficient incorporation of recreational values into forest management planning.

## Footnotes

1 https://www.openstreetmap.org/.
